# Disease Models & Mechanisms announces a new Editor-in-Chief

**DOI:** 10.1242/dmm.048652

**Published:** 2020-12-22

**Authors:** Paresh Vyas

**Affiliations:** MRC Molecular Haematology Unit, MRC Weatherall Institute of Molecular Medicine, University of Oxford, John Radcliffe Hospital, Headington, Oxford OX3 9DS, UK

In 2018, Disease Models & Mechanisms (DMM) Editor-in-Chief Monica Justice asked the Board of Directors of The Company of Biologists to identify and recruit her successor. The DMM Advisory Group, a subset of the Directors of the Company, comprising Sadaf Farooqi, Peter Rigby and myself, joined by two other Directors, Sarah Bray and Matthew Freeman, was tasked with overseeing the recruitment process. After a rigorous selection process, we are delighted to announce that Professor Elizabeth Patton will be the next Editor-in-Chief of DMM.

Liz Patton has a personal chair in Chemical Genetics and is Principal Investigator at the MRC Human Genetics Unit, University of Edinburgh, UK. She graduated with a BSc (Hons) from the University of King's College, Dalhousie University in Halifax, Nova Scotia, and completed a PhD with Mike Tyers studying yeast metabolism and cell cycle at the University of Toronto. Liz then moved into the zebrafish field, to work with Len Zon at Harvard Medical School, where she developed a zebrafish BRAF model for melanoma. Zebrafish models of melanoma have remained the focus of her work ever since. She started her independent career in Edinburgh, at the MRC Human Genetics Unit, where her lab uses genetics and chemical-biology to study fundamental processes in melanocyte development during embryogenesis, and explore how they contribute to melanoma.

Liz is well known in the zebrafish and DMM communities. She has been a DMM Editor since 2013, when she was interviewed for the ‘A Model for Life’ interview series (Patton, 2013). DMM will talk to Liz again in 2021, to find out what she has been doing since. Liz will also outline her vision for the journal in an Editorial to be published early in 2021.

The appointment of Liz followed a lengthy consultation and interview process. We initially asked DMM Editors, Editorial Board members, authors, reviewers and the wider community to provide feedback on DMM, its future and its new Editor-in-Chief. With this information, recruitment consultants Perrett Laver further talked to researchers within the community to identify potential candidates. Applications were invited and received from a broad range of researchers. The Advisory Group shortlisted and remotely interviewed a really strong and international selection of applicants. Liz was the standout candidate. We are so pleased to have Liz on board. She brings vitality and a passion for the remit of the journal, and is deeply embedded in the community. We were excited by her vision and commitment to bring a multidisciplinary community together to help transform the lives of patients with diverse diseases.

The consultation has also given us an opportunity to consult on and identify how we could improve DMM. DMM was launched in 2008 to publish research to better understand, diagnose and treat human disease using model systems. At its core, DMM seeks to provide a forum to promote multidisciplinary collaborative research among basic researchers, translational scientists and clinician scientists.

We learned that DMM provides a much-needed, highly appreciated niche for those working on disease models, with a strong presence in many model organism communities. In particular, you liked the high standards that the DMM Editors set for model organism research, with well-informed decisions on papers, strong editorial and publishing policies, and comprehensive review articles highlighted. You also like DMM's charitable grants and activities, particularly its focus on supporting early-career researchers.

However, we can and will do better. Many commented that they would like to see DMM papers being more highly cited and making more of an impact. Many wanted a stronger focus on disease mechanism and a clearer sense of clinical relevance. Some mentioned the challenge of improving the visibility of DMM in an increasingly competitive environment.

We also asked which areas you would like to see DMM expanding into in the future. Bioengineering (advanced *in vitro* models, three-dimensional tissue culture and organ-on-a-chip models), use of large datasets as a starting point, or as validation tools, for mechanistic studies, immunology and infection (rather presciently given the current pandemic), preclinical data on precision therapies, and rare diseases were all flagged. Liz has taken note of all of the outcomes of this consultation process. We offer our thanks to all those who took the time to participate in the consultation.

Monica continues as Editor-in-Chief until the end of this year, with Liz's appointment beginning on 1 January 2021. However, Liz has already started working with Monica and the editorial team based in Cambridge, UK, to ensure a seamless handover.

It just remains for me to firstly thank Monica on behalf of DMM and the Board of Directors of The Company of Biologists. Monica has been an excellent Editor-in-Chief. She has been selfless in her commitment to DMM and its team. DMM has run incredibly smoothly under her leadership. Finally, I want to congratulate Liz on her appointment. We all are very much looking forward to working with her in this new and exciting chapter for DMM.
